# Anticipatory Regulation of Action Control in a Simon Task: Behavioral, Electrophysiological, and fMRI Correlates

**DOI:** 10.3389/fpsyg.2013.00047

**Published:** 2013-02-12

**Authors:** Gamze Strack, Christian Kaufmann, Stefanie Kehrer, Stephan Brandt, Birgit Stürmer

**Affiliations:** ^1^Department of Psychology, Humboldt-Universität zu BerlinBerlin, Germany; ^2^Department of Neurology, Berlin NeuroImaging CenterBerlin, Germany

**Keywords:** cognitive conflict, cueing, EEG, fMRI, pre-SMA, Simon task, anticipation, cognitive control

## Abstract

With the present study we investigated cue-induced preparation in a Simon task and measured electroencephalogram and functional magnetic resonance imaging (fMRI) data in two within-subjects sessions. Cues informed either about the upcoming (1) spatial stimulus-response compatibility (rule cues), or (2) the stimulus location (position cues), or (3) were non-informative. Only rule cues allowed anticipating the upcoming compatibility condition. Position cues allowed anticipation of the upcoming location of the Simon stimulus but not its compatibility condition. Rule cues elicited fastest and most accurate performance for both compatible and incompatible trials. The contingent negative variation (CNV) in the event-related potential (ERP) of the cue-target interval is an index of anticipatory preparation and was magnified after rule cues. The N2 in the post-target ERP as a measure of online action control was reduced in Simon trials after rule cues. Although compatible trials were faster than incompatible trials in all cue conditions only non-informative cues revealed a compatibility effect in additional indicators of Simon task conflict like accuracy and the N2. We thus conclude that rule cues induced anticipatory re-coding of the Simon task that did not involve cognitive conflict anymore. fMRI revealed that rule cues yielded more activation of the left rostral, dorsal, and ventral prefrontal cortex as well as the pre-SMA as compared to POS and NON-cues. Pre-SMA and ventrolateral prefrontal activation after rule cues correlated with the effective use of rule cues in behavioral performance. Position cues induced a smaller CNV effect and exhibited less prefrontal and pre-SMA contributions in fMRI. Our data point to the importance to disentangle different anticipatory adjustments that might also include the prevention of upcoming conflict via task re-coding.

## Introduction

Our ability to exert cognitive control in order to adjust ongoing performance to changing environmental conditions is essential for flexible behavior in everyday life. Whenever prior information or experience is available we attempt to avoid costs of inappropriate behavior (Kool et al., 2010). Such anticipatory processes are especially inevitable in settings calling for online control: the need for cognitive control elicited by preceding difficulties or errors engenders regulatory processes and people build subjective predictions about upcoming task demands on the basis of accumulating task knowledge. As such, cognitive control adjustments should be generally viewed along an anticipatory-online control continuum (Ullsperger and King, 2010). Yet, almost everything we know about action control refers to instantaneous online mechanisms like conflict detection and resolution. In contrast, little is known about the anticipation of these processes. Anticipatory regulation is a broad term and includes types of anticipatory control that may extensively differ in function. Recently, Ridderinkhof et al. (2010) conceptualized anticipatory regulation along two dimensions. The first dimension describes the point in time that triggers anticipatory regulation: *Reactive* anticipatory regulation is prompted by prior behavior or events such as preceding errors or cognitive conflicts. As well, anticipatory control regulation can be induced by prior information such as cues and is then of genuine *prospective* nature. The second dimension describes different types of adjustments that may accomplish both prospective as well as reactive anticipatory regulation: *Proactive*^1^ adjustments may boost subsequent online conflict control by modifying the level of response or inhibition readiness. Or, alternatively, *preemptive* adjustments may to diminish or avoid the need for cognitive control by modifying the level or focus of selective attention. Such adjustments are obviously not only thinkable in the context of cognitive conflict but in all situations with high cognitive demand or increased error probability. Wühr and Kunde (2008) presented an excellent example of preemptive regulation in a Simon conflict task (Simon, 1967). In this task, spatially oriented responses are assigned to a non-spatial stimulus feature (e.g., stimulus figure). The task-irrelevant stimulus location, however, alters randomly and either matches or mismatches response location resulting in compatible and incompatible trials. Wühr and Kunde (2008) cued the compatibility of upcoming trials and showed that participants effectively used the actually task-irrelevant stimulus location instead of the task-relevant stimulus feature for response selection. Participants, therefore, changed their attentional focus. The aim of the present study was to further investigate prospective anticipatory regulation in a Simon task by using behavioral, electrophysiological, and hemodynamic measures.

Anticipatory regulation has previously been shown to improve behavioral performance in many situations involving cognitive conflict (Fassbender et al., 2006; Luks et al., 2007; Sohn et al., 2007; Aarts et al., 2008; Donohue et al., 2008; Alpay et al., 2009). Anticipatory processes can also be reflected by an electrophysiological measure, namely the contingent negative variation (CNV; Leuthold et al., 2004). This event-related potential (ERP) is observed during expectancy of an upcoming event (Walter, 1964). The terminal phase of the CNV prior to target onset reflects general preparation with sensory, motor, and cognitive shares depending on the particular task (e.g., Damen and Brunia, 1994; Fan et al., 2007). Yet, few CNV studies investigated the influence of higher-level processes like the anticipation of looming conflict. Fan et al. (2007) showed that cues eliciting higher unspecific response readiness enhanced the CNV amplitude in a conflict paradigm. We showed in a previous study that the CNV is susceptible for both cue-induced prospective anticipation and reactive anticipation due to the trial sequence (Alpay et al., 2009). More is known about post-target ERPs that indicate processes of online action control such as the N2 (Folstein and van Petten, 2008 for a review). This ERP deflection is a negative wave with a fronto-central distribution that usually peaks between 200 and 350 ms after onset of the imperative stimulus. The amplitude of the anterior N2 is magnified by processes involving cognitive control (Kopp et al., 1996; Heil et al., 2000; Nieuwenhuis et al., 2003; Falkenstein, 2006; Kehrer et al., 2009). The N2 has been associated with activation of the anterior cingular cortex (ACC), a ventrally located area within the posterior medial frontal cortex (pMFC; Nieuwenhuis et al., 2003; Ridderinkhof et al., 2004). The seminal conflict monitoring theory postulates that the ACC detects conflict and calls for top-down control processes to resolve concurrent response tendencies (Botvinick et al., 2004). There are several functional neuroimaging functional magnetic resonance imaging (fMRI) studies that provide evidence for an association of pMFC activation and online action control (Ridderinkhof et al., 2004 for a review). Rather few fMRI studies investigated *anticipatory* regulation of online action control. Some of them focused on the ACC and found respective anticipatory activation after explicit cueing of upcoming control demands (Sohn et al., 2007; Aarts et al., 2008). Anticipatory processes in these studies were of proactive nature and it remains an open question whether ACC activation can also be expected in preemptive anticipatory adjustments. The literature provides inconclusive results about the role of the ACC in anticipatory regulation since some studies did not find any preparatory ACC activation (MacDonald et al., 2000; Fassbender et al., 2006; Luks et al., 2007; Donohue et al., 2008). Another candidate region that might be also involved in anticipatory regulation of action control is the pre-SMA. Mars et al. (2009) and Neuhaus et al. (2010) assume that pre-SMA rather than ACC activation is associated with situations involving direct competition (Ullsperger and von Cramon, 2001), inhibition (Nachev et al., 2007), updating (Shima et al., 1996), or reprogramming (Isoda and Hikosaka, 2007) of actions. Using model-based fMRI that takes individual differences into account, Forstmann et al. (2008a) reported that the Response time (RT) distribution of response capture covaried with pre-SMA activation. Some researchers claim a key role for the pre-SMA in anticipatory control regulation. Hikosaka and Isoda (2010) concluded in their review that pre-SMA activation occurs when cues indicate a switch, whereas ACC activation occurs after error feedback. Ullsperger and King (2010) seized this idea, proposing that not only task switching but rather all processes of online action control can be more or less regulated by anticipation, and that underlying processes might be associated with pre-SMA activation.

In the present study we cued upcoming control demands in a Simon task in order to investigate how participants anticipate and which neural structures are associated with this anticipatory regulation. In particular, we were interested in whether these processes are performed by the ACC or the pre-SMA. Therefore, we employed a Simon task and presented one of three cue types prior to each Simon target that either (1) informed about the compatibility of the upcoming Simon target (rule cues), or (2) informed about the spatial position of the upcoming Simon stimulus (position cues), or (3) provided no information (non-informative cues). Rule cues were expected to induce prospective anticipatory regulation of action control and thus to be most beneficial for task completion. In contrast, position cues were assumed to trigger an anticipatory attentional shift to the correct stimulus location. Both rule and position cues reduced the stimulus set twofold while keeping the response set bivalent, i.e., no prediction of the response key was possible. Non-informative cues were introduced as a control condition that neither reduced the stimulus nor the response set. We additionally applied NoGo^2^ trials in order to prevent preemptive adjustments such as the deduction of the correct response from the stimulus position (e.g., “compatible” means to press the key corresponding to the stimulus location). Such preemptive adjustments were indicated by behavioral measures in a cued Simon task in Wühr and Kunde (2008): participants shifted their attention from the task-relevant stimulus figure to the task-irrelevant stimulus position (e.g., a cue indicating an incompatible trial means a crossed response). We investigated additional measures that are indicative of conflict, e.g., the N2 and conditional accuracy functions (CAFs) in order come to a better understanding of the underlying adjustments in the present study. CAFs plot behavioral accuracy as a function of RT speed and usually show that fast responses tend to be more error-prone, especially in incompatible conditions (response capture, Ridderinkhof et al., 2010 for a review). Typically accuracy starts low and improves quickly within the fastest segment of RTs. According to Ridderinkhof et al. (2010) the slope between the first two bins in a CAF indexes the overcoming of response capture in incompatible trials (the steeper, the more response capture, that is, the more conflict). If rule cues lead to preemptive adjustments (circumvention of the original instruction) they should be associated with a lower N2 amplitude and less indication of response capture. Non-informative cues, since being a measure of the unmodified Simon effect, were assumed to show the opposite pattern. Otherwise, if rule cues just modulate response readiness (proactive adjustments) the N2 should compare to that after non-informative cues. They were also expected to exhibit magnified N2 amplitudes for incompatible events (as compared to compatible events). By contrast, a N2 compatibility effect should be absent after rule cues if they trigger preemptive anticipatory regulation despite our NoGo manipulation. In order to get more insight into the neural basis of prospective anticipation we investigated electrophysiological and hemodynamic measures of pre-target processes. We expected rule cues to enhance the anticipatory pre-target CNV. At the hemodynamic level, we were interested in the neural networks that accomplish anticipatory regulation. We expected that proactive preparation to upcoming conflicts should be associated with ACC activation as reported in studies that investigated this type of anticipatory regulation (Sohn et al., 2007; Aarts et al., 2008). However, there is little research about networks involved in preemptive adjustments. One important candidate structure in such networks might be the pre-SMA because rule cues trigger the retrieval of relevant response contingencies and, thus, prospectively prepare for action selection (Rushworth, 2008; Ullsperger and King, 2010).

## Materials and Methods

### Participants

Thirty-nine students participated in a first EEG session, whereas the data of eight participants had to be excluded afterward (six due to augmented ocular or movement-related artifacts, one due to extremely slow responses, one due to technical problems). The remaining 31 participants took part at a second session where fMRI was measured. One participant was excluded from further analysis because of incomplete behavioral data acquisition during the fMRI session. Two participants were excluded because of movement artifacts. All of the remaining 28 students (21 women, 7 men; 18–31 years old, mean age = 22.6 years) had normal or corrected-to-normal vision, were right-handed and reported no history of neurological, psychiatric, or major medical disorder. All participants were students from the Humboldt-Universität zu Berlin that either received course credits or were paid 8 EURO per hour for volunteering at the experimental procedures. They signed an informed consent prior to both experimental sessions. All procedures were previously approved by the ethical review board at the Charité university medical center.

### Procedure, stimuli, and design

Participants completed a first EEG session and, 4–6 weeks later, a second fMRI session. In the EEG session, participants were seated in a sound-attenuated chamber at a constant viewing distance of 100 cm to a 17″ TFT computer screen. They responded by pressing one of two response keys horizontally arranged on a table (distances: 20 cm to participant, 30 cm between keys). The timing of the task program that displayed stimuli and recorded behavioral responses was triggered every 2 s by simulated scanner main pulses, i.e., timing of the experiment was exactly the same during the EEG and fMRI sessions. In the fMRI session participants lay supine in the MR scanner. Imaging data were collected using a standard birdcage head coil. Vacuumed pillows were used to minimize head movements. Stimuli were projected on a screen mounted above the MRI head coil and could be viewed through an attached mirror. The main pulses of the scanner determined the timing of the task program that displayed stimuli and recorded behavioral responses (Presentation^®^ software)^3^. Participants responded with their left or right index finger by pressing one of two optical response keys placed at their hands.

**Figure 1:**
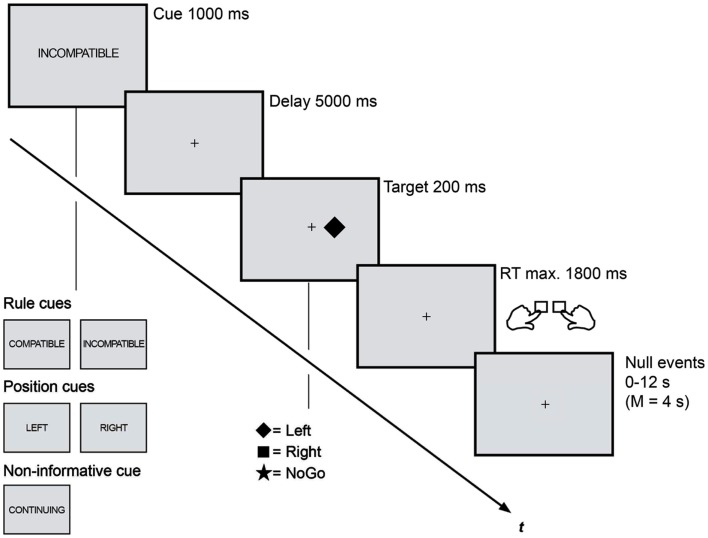
**Rule cues predicted compatibility and were for this reason expected to trigger anticipatory action control**. These cues reduced the task set from four to two possible S-R assignments and were presented along two different control conditions: position cues that predicted the upcoming stimulus position and reduced the task set in the same amount enabled an attentional shift to the task-relevant visual half. Non-informative cues did not induce any anticipatory processes and lead not to a task set reduction. Trial procedure as well as timing was kept identical in the EEG and fMRI session.

The trial procedure was identical in both sessions (Figure 1). Each trial consisted of a cue period (1 s), a delay period (5 s), a stimulus period (0.2 s), and a fixed time interval for the response (1.8 s, whereas responses later than 1 s were classified as too late). Stimulation was presented white on a dark gray background on a flat computer screen in the EEG chamber and via a back-projection screen in the MRI scanner. A white fixation dot (0.09° visual angle) marked the center of the screen as long as no cue stimulus was displayed.

A horizontal Simon task was combined with three different types of precues: rule cues (RULE), position cues (POS), and non-informative cues (NON). (1) RULE cues (“compatible,” “incompatible”) informed about the compatibility of the upcoming Simon task and, thus, were assumed to enable the anticipation of the subsequent control demand. (2) POS cues informed about the spatial location (“left,” “right”) of the upcoming Simon stimulus and enabled the anticipatory allocation of spatial attention. (3) A fifth cue was non-informative (“continue”) and served as control condition that provides no information for preparatory processes. Cue stimuli were centrally displayed as German translations of the capitalized words compatible (“KOMPATIBEL”), incompatible (“INKOMPATIBEL”), left (“LINKS”), right (“RECHTS”), and continue (“WEITER”). The cues subtended a visual angle of 1° − 2.5° in horizontal and 0.23° in vertical orientation. Each Simon trial was randomly cued with one of these cues that were presented with equal probabilities. All cues were valid and the cue-target combinations were counterbalanced. Participants were asked to prepare for the subsequent Simon task by using the cue information as good as possible.

After the cue delay participants were shown one of three Simon stimuli that were randomly presented either left or right (0.5° visual angle) of fixation: one indicated a right-hand response, the second indicated a left-hand response, and the third was a NoGo. A white-filled square or diamond served as Simon Go stimuli, while a star indicated a NoGo (each 0.75° visual angle). The NoGo condition was randomly presented in 1/3 of all trials, resulting in equal probabilities for the occurrence the three stimuli. Participants were instructed to respond as fast and as accurate as possible.

Both experimental sessions consisted of blocks each containing 75 trials in a pseudorandom sequence (lasting approximately 13 min). Participants performed six blocks in the EEG session (450 trials, approximately 1.5 h recording time). In the fMRI session they completed four blocks (300 trials, approximately 55 min scanning time) each recorded as one run. That is, every cue (“KOMPATIBEL,” “INKOMPATIBEL,” “LINKS,” “RECHTS,” “WEITER”) was presented 90 (EEG) or 60 (fMRI) times while the subsequent stimulus type was either compatible, incompatible, or a NoGo with equal probabilities (no false RULE cues). We reduced the total amount of trials in the fMRI session to keep the scanning duration feasible. Opportunity for brief rest was given between blocks in both sessions. The trial order within every block was optimized with an algorithm designed to maximize the separability of the conditions in a rapid event-related fMRI design (optseq2; Dale, 1999). After the 2-s intertrial interval, period of fixation lasting between 0 and 12 s, jittered in increments of 2 s (mean = 4 s), were interleaved with the experimental trials as determined by the optimization program. The order of runs and the stimulus-to-response assignment were counterbalanced among sessions and participants. Participants completed one 75-trial practice block prior to both sessions.

### Data analyses

For all analyses trials with erroneous responses, trials immediately following errors and responses faster than 100 ms or slower than 1000 ms after target onset were discarded (RT, EEG) or modeled separately (fMRI). This reduced ERP data by 4.6% and fMRI data by 3.9%. NoGo targets were excluded from all analyses except for the behavioral analysis of false alarms. For RT and EEG analyses, ANOVAs are Huynh Feldt-corrected and *post hoc* comparisons Bonferroni-corrected. *T*-tests are two-tailed, if not mentioned otherwise.

### Behavioral data

For RT distributional analysis RTs for each cue condition (RULE, POS, NON) and target condition (compatible, incompatible) were rank-ordered and divided into quartiles (four equal-sized bins). Mean RTs and accuracies for each condition and each quartile were computed. Conditional accuracy plots were created for each of the three cue conditions by plotting the accuracy of mean RTs for incompatible trials on the *y*-axis as a function of response speed on the *x*-axis (mean RTs for both compatibility conditions in quartiles). Slopes were calculated for the three delta plot segments determined by the data points of quartile 1 and 2 (slope 1), quartile 2 and 3 (slope 2), and quartile 3 and 4 (slope 3). ANOVAs conducted involved the factors cue (RULE, POS, NON) and slope (slope 1, slope 2, slope 3).

### EEG recording and analysis

EEG was continuously recorded at 64 Ag/AgCl electrodes in an extended 10–20 system montage referenced to the participants’ left mastoid. AFz served as ground electrode. The horizontal electrooculogram (EOG) was recorded from the outer canthi and vertical EOG was recorded from FP1 and below the left eye. All electrode impedances were kept below 5 kΩ. The EEGs and EOGs were recorded DC at a sampling rate of 1000 Hz and filtered online using a 250-Hz high cut-off. After recording the EEG was down-sampled offline to 250 Hz. Electrophysiological signals were recorded with Brain Vision Recorder and analyzed with Brain Vision Analyzer (Brain Products GmbH, Germany).

For the CNV analysis the signals were filtered offline with an additional low-pass filter of 5 Hz, 48 dB/oct. Artifacts with voltage steps exceeding 20 μV per sampling point were automatically removed. Cue-locked epochs of 9 s were created for each trial, starting 1 s before cue onset and ending 2 s after target onset. A time period of 1 s before cue onset was subtracted as baseline. Segments with amplitudes exceeding ±200 μV were automatically discarded from further analysis. In addition, trials were visually inspected and discarded if ocular artifacts occurred in the last 1 s before target onset (time interval of interest). The EEG epochs were averaged separately for each participant and cue condition. We analyzed the late CNV in a 1-s time interval immediately before target onset. An ANOVA was conducted containing 60 EEG electrodes and three cue types (RULE, POS, NON). A *post hoc* ANOVA additionally tested for compatibility after RULE cues (RULE prediction × electrode).

For the N2 analysis EEG was low-pass filtered with 30 Hz and high-pass filtered with 1 Hz, each with 48 dB/oct (time constant = 0.1592 s). Recorded signals were automatically removed when voltage steps exceeded 50 μV per sampling point, as well as when the difference between maximal and minimal activity fell below 0.50 μV within a 100-ms interval. All EEG channels were then submitted to an Infomax independent component analysis (ICA) algorithm for blink-correction. The ICA component reflecting an eye blink was identified for each subject excluded from signal synthesis of ICA sources to EEG channels. Other artifacts were eliminated semi-automatically. Target-locked segments of 9 s were created for each trial, starting 1 s before cue onset and ending 2 s after target onset. EEG epochs were averaged separately for each participant and each cue and compatibility condition. Since a pre-target baseline might be biased by cue-induced effects, we analyzed the N2 independently of a baseline following a peak-to-peak approach that has been introduced by Nieuwenhuis et al. (2003). Accordingly, peak-to-peak detection was determined for every condition in each participant in the Fz electrode (Nieuwenhuis et al., 2003): the N2 peak was automatically identified within time windows of 200–450 ms after stimulus onset. The N2 amplitude was then defined as the amplitude of this peak minus the immediately preceding positive peak (P2). Automatic peak detection was additionally visually inspected and corrected, if necessary. The ANOVA contained three cue types (RULE, POS, NON) and two Simon trial compatibilities (compatible, incompatible).

### fMRI data acquisition and analyses

Data were acquired at the Berlin NeuroImaging Center (Germany) on a 1.5-T MR scanner equipped with a circular-polarized head coil (Siemens Sonata, Erlangen, Germany) with an T2*-weighted single-shot gradient echo planar imaging sequence: 35 slices (interleaved), 3 mm isotropic resolution, 64 × 64 matrix, FOV = 192 mm, TE = 40 ms, TR = 2.00 s, flip angle = 90°, 1640 AC-PC oriented images for each run. Before functional runs, 176 anatomical T1-weighted slices were acquired (spatial resolution 1 mm × 1 mm × 1 mm, TR = 12.24 ms, TE = 3.56 ms, flip angle = 23°, 256 × 224 matrix; Deichmann, 2005). A vacuum head cushion was used to immobilize the participants’ heads and necks in order to reduce movement artifacts. Earplugs were provided to attenuate background noise and additional headphones were used to communicate with subjects. Image preprocessing and analysis was carried out with SPM5 (Statistical Parametric Mapping)^4^. The first four volumes of each functional time series were discarded to avoid non-steady state effects caused by T1 saturation. Subsequently, motion correction estimation revealed that no subject showed more than 2 mm head movement (translation) and more than 1° of rotation during one run. After slice time correction of the functional data the anatomical data set was co-registered with the mean T2* image and T1-weighted images were segmented into gray matter, white matter, and cerebrospinal fluid. The gray matter of the co-registered structural image was spatially normalized to the standard template provided by the Montreal Neurological Institute (MNI) template using an automated spatial transformation (12-parameter affine transformation followed by non-linear iterations using 7 × 8 × 7 basis functions). The resulting transformation matrix was applied to the T2* data, and a resampling to a resolution of 3 mm × 3 mm × 3 mm voxel size was performed. Finally, the normalized images were smoothed with a Gaussian kernel (full width at half maximum) of 9 mm to create a locally weighted average of the surrounding voxels.

Statistical analyses were performed with a general-linear model approach. First, each subject was modeled independently. Five vectors of event onsets were created for model estimation, defining the experimental conditions of RULE cues (compatible, incompatible), POS cues (left, right), and NON-cues (continue). These pre-target effects were calculated locked to the cue onset. Additionally, error and post-error trials were modeled as one separate condition, although their quantity was insufficient for further analysis. The regressors were then convolved with a canonical hemodynamic response function (HRF) and employed as event-related regressors to model the BOLD responses within each experimental block. The HRF was combined with a temporal derivative as we assumed the peak response to vary in time. Six spatial realignment parameters served as additional regressors to remove signals correlated with head motion. Slow signal drifts were removed with a high-pass filter cut-off of 128 s. Model parameters were estimated using classical restricted maximum likelihood estimates. The estimation was made including a first-order autoregressive model in order to estimate temporal autocorrelations in the time series data and to correct for non-sphericity by adjusting the degrees of freedom appropriately. Voxelwise statistical parametric maps (SPM) were calculated for linear contrasts between regressors of interest for each subject. The resulting contrast images were submitted into a group analyses, treating subjects as random effects. Whole brain statistics were calculated for the contrasts RULE cue > NON-cue, POS cue > NON-cue, and RULE cue > POS cue (and their respective reversed contrasts) by performing one-sample *t*-tests. We additionally tested for effects of compatibility in RULE cues, RULE incompatible > RULE compatible. All results reported relate to activation averaged across voxels in clusters larger than 25 contiguous voxels meeting a threshold at *p* < 0.05, corrected for multiple measurements (FDR; Genovese et al., 2002). All resulting cluster maxima were converted to Talairach space^5^ and entered into the Talairach Damon (Lancaster et al., 2000) in order to determine the nearest anatomical loci. Pearson correlations between individual cluster activation in all frontal areas (*Z*-standardized beta-values) and the behavioral RULE cue benefit (*Z*-standardized NON-RULE RT difference) were calculated.

## Results

### Behavioral results

**Figure 2:**
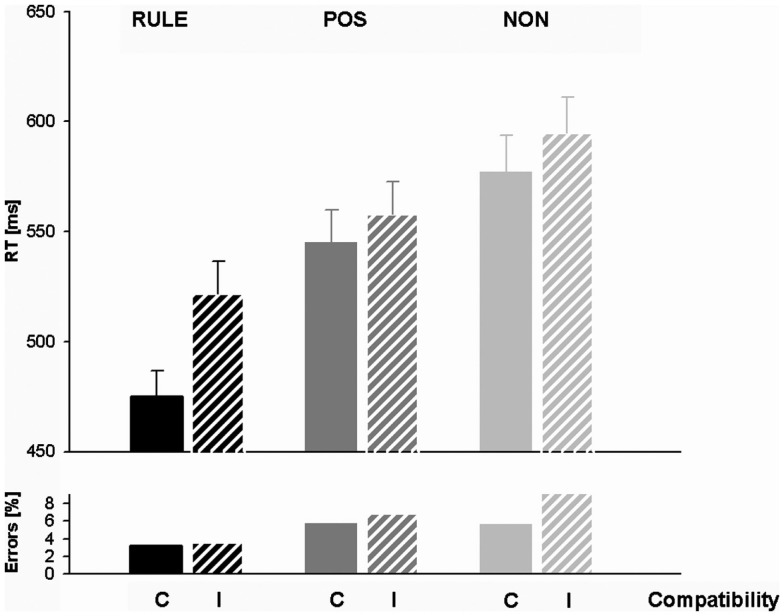
**Response times (RTs) and error percentages for rule cues (RULE), position cues (POS), and non-informative cues (NON) in compatible and incompatible trials**. Note the particularly short RTs for rule cued compatible trials.

**Figure 3:**
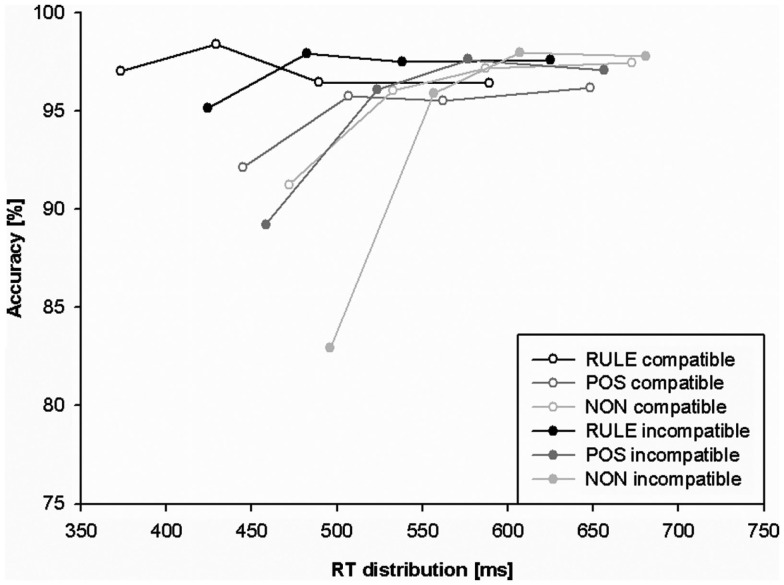
**Conditional accuracy function (CAF) plotting behavioral accuracy as a function of response speed for rule cues (RULE), position cues (POS), and non-informative cues (NON)**. The slope of the fastest portion (first segment) of RTs is conceived as a measure of response capture.

Response times and accuracy data (error percentages) for all factor levels are displayed in Table 1 and Figure 2. Figure 3 shows the effect of accuracy as a function of RT dispersion.

**Table 1:** **Means and standard deviations (SDs) of reaction times (RT) and percentages of error (PE) as a function of the factors compatibility (compatible, incompatible) and cue condition (RULE, rule cues; POS, position cues; NON, non-informative cues)**.

	RULE		POS		NON	
	RT	PE	RT	PE	RT	PE
**COMPATIBLE**						
Mean	474.9	3.2	545.3	5.8	577.0	5.6
SD	66.1	3.0	81.2	4.0	93.2	5.3
**INCOMPATIBLE**						
Mean	521.6	3.6	557.8	6.9	595.1	9.2
SD	83.9	2.8	83.1	4.0	90.4	5.2

#### Response times

An exploratory ANOVA containing both sessions revealed a non-significant trend between the sessions, *F*s < 3.68, *p*s > 0.06. We, therefore, collapsed data across sessions. A typical Simon effect of 26 ms, *F*(1,27) = 35.0, *p* < 0.001, and a main effect of cue condition occurred, *F*(2,54) = 70.78, *p* < 0.001. RULE cues (*M* = 498 ms, SD = 74) enabled by 88 ms faster responses than NON-cues (*M* = 586 ms, SD = 90), *t*(27) = 9.41, *p* < 0.001, and by 53 ms faster than POS cues (*M* = 552 ms, SD = 81), *t*(27) = 7.05, *p* < 0.001. POS cues still triggered an RT benefit of 34 ms against NON-cues, *t*(27) = 7.40, *p* < 0.001. We calculated *t*-tests in order to examine whether these cueing benefits were present in both compatibility conditions: RULE against NON-was faster in compatible assignments, *t*(27) = 9.44, *p* < 0.001 as well as in incompatible assignments, *t*(27) = 8.0, *p* < 0.001, and POS versus NON-was also faster in both compatible, *t*(27) = 4.67, *p* < 0.001, and incompatible trials, *t*(27) = 8.35, *p* < 0.001. An overall interaction of compatibility and cue condition, *F*(2,54) = 14.82, *p* < 0.001, indicated a particularly pronounced RT difference between the compatibility conditions after RULE cues, *t*(27) = 7.71, *p* < 0.001. However, the compatibility effect was also significant for trials with POS cueing, *t*(27) = 2.8, *p* < 0.05, and for trials with NON-cueing, *t*(27) = 2.66, *p* < 0.05. In sum, RTs showed utilization of both RULE and POS cues since they were fastest after RULE cues, intermediate after POS cues, and slowest after NON-cues. In addition, compatibility effects were present in all cueing conditions while they were enhanced after RULE cues. However, this greater compatibility effect came about because RULE cues speeded up compatible assignments relatively more than incompatible trials (and not because incompatible responses were slowed down).

#### Accuracy

The overall accuracy in the EEG session (6.12%) did not differ from that in the fMRI session (5.33%), *F*(1,27) = 1.69, *p* = 0.20. For this reason, data were collapsed across sessions. About 2.94% of NoGo trials were false alarms that were analyzed separately for cue effects. This analysis revealed that false alarms occurred more often after RULE cues than both after POS cues, *t*(27) = 4.96, *p* < 0.001, and after NON-cues, *t*(27) = 5.13, *p* < 0.001. The false alarm rate between POS and NON-cues did not differ, *t*(27) = 1.65, *p* > 0.1. Thus, the false alarm rate suggested that the NoGo manipulation that was introduced to prevent preemptive adjustments was less effective after RULE cues. Accuracy was further analyzed for all Simon go trials with a cue (RULE, POS, NON) × trial compatibility (C, I) within-subjects ANOVA. A main effect of cue condition, *F*(2,54) = 20.98, *p* < 0.001, indicated that trials with RULE cues entailed less errors (3.41%) than both trials with NON-cues (7.44%), *t*(27) = 5.48, *p* < 0.001, and trials with POS cues (6.32%), *t*(27) = 6.03, *p* < 0.001. The numerically higher accuracy for trials cued with POS cues against trials cued with NON-cues failed significance, *t*(27) = 1.64, *p* = 0.11. A main effect of compatibility, *F*(1,27) = 5.70, *p* < 0.05, was due to more errors in incompatible trials (6.56%) than in compatible ones (4.89%). The factors cue condition and compatibility interacted, *F*(2,54) = 5.35, *p* < 0.01, since compatibility affected accuracy only in trials with NON-cues, *t*(27) = 3.14, *p* < 0.01, but not in the other cue conditions, *t*s < 1.1. In sum, trials with RULE cues were accomplished most accurate and exhibited no compatibility effect in accuracy rates. The accuracy results therefore support the former notion that the higher compatibility effect in RTs for RULE cues might rather originate from a relatively greater cue benefit for compatible than for incompatible trials and not from a greater cognitive conflict (that would be associated with slower responses and lower accuracy). As opposed to this, trials with NON-cues showed typical conflict effects with lowered accuracy and a Simon effect in accuracy rates.

We analyzed accuracy as a function of RT dispersion in CAFs to investigate whether prospective anticipation induced by cues diminishes the impact of the misleading stimulus location. The strength of initial response capture in the Simon task is reflected in the frequency of fast errors that are thought to indicate stronger initial capture (Ridderinkhof et al., 2010 for a review). The slope value between the two fastest RT segments indexes the strength of initial response capture. We predicted that RULE cues should be associated with least response capture while NON-cues should be associated with most response capture. We submitted the slope between the first two fastest segments of the CAF to a repeated-measures ANOVA including the factors cue condition and compatibility. Overall main effects were present for cues, *F*(2,54) = 21.04, *p* < 0.001, as well as for compatibility, *F*(1,27) = 22.32, *p* < 0.001. The main effect for compatibility was induced by a steeper positive-going CAF slope for incompatible than compatible trials, *t*(27) = 4.7, *p* < 0.001 (indicating more response capture). The main effect of cue condition was due to a steeper positive slope for trials with NON-cues than for trials with RULE cues, *t*(27) = 6.24, *p* < 0.001, as well as for trials with NON-cues than trials with POS cues, *t*(27) = 2.88, *p* < 0.05. The slope of trials with POS cues was also more positive-going than the slope of events with RULE cues, *t*(27) = 4.067, *p* = 0.001. An overall interaction of compatibility and cue condition, *F*(2,54) = 12.85, *p* < 0.001, indicated that the latter differences between cue conditions did not occur in compatible trials, *t*s(27) < 2.2, *p*s > 0.1, but rather in incompatible trials: *t*(27) > 3.39, *p*s < 0.01. Compatibility effects in CAF slopes were strongest for NON-trials, *t*(27) = 5.37, *p* < 0.001, while weaker in POS trials, *t*(27) = 2.57, *p* = 0.05, and not significant in RULE trials, *t*(27) = 1.36, *p* > 0.5. Taken together, response capture and cognitive conflict, respectively, as indicated by CAF slopes were only present in incompatible trials that exhibited strongest effects for NON-cues, intermediate effects for POS cues, and smallest effects for RULE cues.

### Electrophysiological results

**Figure 4:**
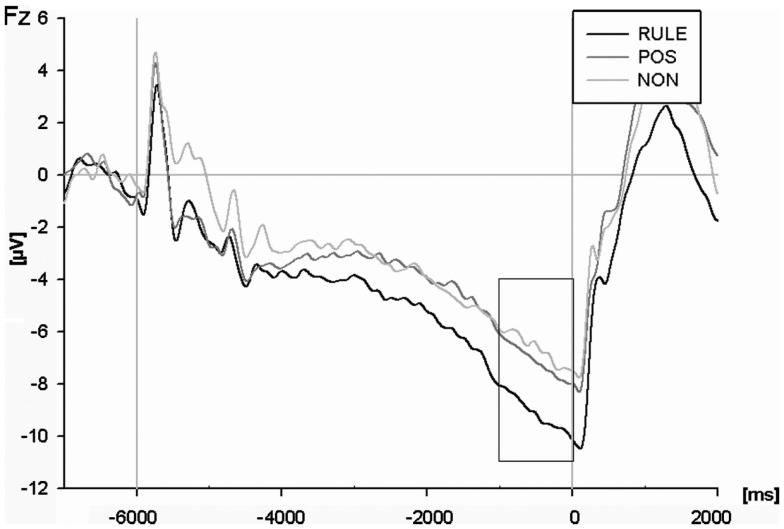
**The contingent negative variation (CNV) at the Fz electrode for rule cues (RULE), position cues (POS), and non-informative cues (NON)**. The analysis refers to the terminal second before target onset.

An ANOVA of the cue-locked CNV amplitude (see Figure 4) including all electrodes and cue types revealed an interaction of electrode and cue condition for the time window 1 s before target onset, *F*(118,3186) = 4.09, *p* < 0.001. Additional ANOVAs each comparing two cue conditions showed that RULE cues elicited a greater CNV compared to NON-cues across all electrodes, *F*(59,1593) = 5.62, *p* = 0.001. RULE cues also generated overall more negativity compared to POS cues, *F*(59,1593) = 3.70, *p* < 0.01. The CNV amplitude for NON-was less pronounced than for POS, *F*(59, 1593) = 2.26, *p* < 0.05. A *post hoc* *t*-test for compatibility was calculated solely for RULE cues because only in this condition compatibility was predicted prior target onset: this test revealed no compatibility effect in the anticipatory CNV after RULE cues, *F* < 1.

**Figure 5:**
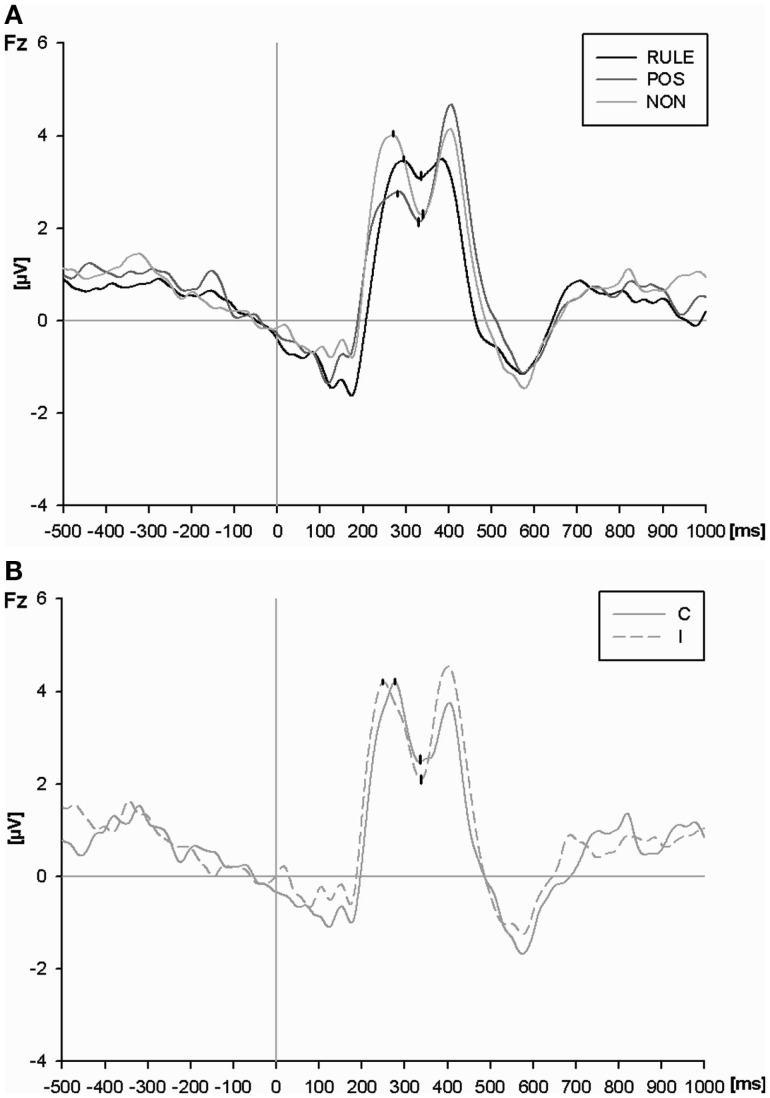
**(A)** The N2 at the Fz electrode for rule cues (RULE), position cues (POS), and non-informative cues (NON) averaged across compatibility conditions. P2 and N2 peaks as determined by automatic peak detection are highlighted. **(B)** The P2 and N2 peaks at the Fz electrode for non-informative cues (NON) plotted separately for compatible and incompatible events.

An ANOVA of N2 at the Fz electrode (Figure 5A) resulted in a main effect of cue condition, *F*(2,54) = 12.62, *p* < 0.001. RULE cues reduced the magnitude of the N2 as compared to NON-cues, *t*(27) = 4.30, *p* < 0.001, and as compared to POS cues, *t*(27) = 4.13, *p* < 0.001. POS cues reduced the N2 amplitude as compared to NON-cues numerically, however, this effect failed significance, *t*(27) = 1.83, *p* = 0.08. The main effect of compatibility, *F*(1,27) = 2.05, *p* = 0.16, as well as the interaction between compatibility and cue condition were not significant, *F*(2,54) = 2.29, *p* = 0.11. In order to test our *a priori* hypothesis that incompatible trials should provoke a larger N2 amplitude than compatible trials, we ran a *t*-test. Such a compatibility effect was present in trials with NON-cues, *t*(27) = 2.98, *p* < 0.01, while it was absent in trials with both informative cue types, *F*s < 1 (see Figure 5B).

In sum, the pre-target CNV amplitude mirrored cue utilization since it was largest with RULE cues, intermediate with POS cues, and least pronounced with NON-cues. Interestingly, the results were reversed for the subsequent conflict-related N2 magnitude (however, the N2 after POS cues was numerically intermediate but differed not significantly from NON-cues). In addition, a compatibility effect was only present in the N2 after NON-cues as expected. Thus, electrophysiological results show conflict indication for NON-cues but not for RULE cues.

### fMRI results

Since we were interested in differentiating networks that are associated with particular cue information we ran pairwise *t*-tests for the activation following the cue onset. Contrasts comparing such anticipatory activation were *RULE cue* > *NON-cue* (results in Table 2), *POS cue* > *NON-cue* (Table 3), and *RULE cue* > *POS cue* (Table 4). We also calculated a contrast between rule cues predicting incompatible and those predicting compatible trials, *RULE incompatible* > *RULE compatible*.

**Table 2:** **Maxima of activation beginning with the onset of rule cues versus non-informative cues activation maxima**.

Anatomical area	Cluster size	Hemisphere	*x*	*y*	*z*	*Z* value
**RULE CUE > NON-CUE**						
Middle frontal G. (BA 10)	47	L	−26	46	8	3.70
Middle frontal G. (BA 10)	37	L	−34	45	21	3.06
Middle frontal G. (BA 9)		L	−34	33	23	2.76
Precentral G. (BA 6)	1070	L	−27	−12	49	5.86
Precentral G. (BA 6)		R	23	−15	49	5.11
Pre-SMA/medial frontal G. (BA 6)		R	7	5	53	4.90
Putamen	456	L	−23	4	7	4.73
Inferior frontal G. (BA 44)		L	−46	0	17	4.42
Putamen	141	R	24	15	1	4.02
Caudate		R	15	2	18	3.14
Thalamus		R	10	−2	7	2.99
Thalamus	26	R	7	−24	−1	4.25
Middle temporal G.	28	L	−54	−35	−3	3.02
Superior temporal G. (BA 22)		L	−46	−38	5	2.81
Inferior parietal L. (BA 40)	795	L	−35	−44	37	5.30
Superior parietal L. (BA 7)		L	−30	−59	44	4.93
Superior parietal L. (BA 7)	437	R	32	−57	48	4.91
Supramarginal G. (BA 40)		R	40	−39	36	4.37
Inferior occipital G. (BA 17)	432	L	−15	−91	−10	4.61
lingual G. (BA 17)		R	15	−83	−1	4.04
Lingual G. (BA 18)		L	−15	−79	−9	4.02
Middle occipital G. (BA 19)	56	R	29	−87	7	3.87
**NON-CUE > RULE CUE**						
Medial frontal G. (BA 10)	91	R	2	56	18	4.07
Superior frontal G. (BA 9)		R	4	55	28	3.82
Superior frontal G. (BA 8)	66	R	21	28	48	5.28
Precuneus (BA 31)	359	R	7	−49	29	4.74
Cingulate G. (BA 31)		L	−2	−36	41	4.03
Anterior cingulate G. (BA 32)		R	2	43	3	3.18
Amygdala	31	R	21	−3	−12	4.88
Parahippocampal G. (BA 35)	96	L	−29	−28	−18	5.31

*Results are reported in Talairach coordinates for peak voxel activations after a False Discovery Rate (FDR) correction (*p* < 0.05; minimum size of each cluster was 25 contingent voxels). Indented rows indicate subordinate clusters. Hemispheres: R (Right) or L (Left); Abbreviations: BA, Brodmann Area; G, Gyrus; L, Lobule*.

**Table 3:** **Maxima of activation beginning with the presentation of position cues versus non-informative cues**.

Anatomical area	Cluster size	Hemisphere	*x*	*y*	*z*	*Z* value
**POS CUE > NON-CUE**						
Putamen	27	L	−23	3	12	3.47
Precentral G. (BA 6)	182	L	−24	−12	49	4.24
Precuneus (BA 7)	164	R	18	−66	50	4.49
Superior parietal L. (BA 7)	255	L	−19	−63	52	4.34
Middle occipital G. (BA 19)	1620	R	26	−84	8	5.36
Lingual G. (BA 18)		L	−12	−85	−12	5.32
Cerebellum (declive)		L	−23	−76	−17	5.06
**NON-CUE > POS CUE**						
No suprathreshold clusters						

*Results are reported in Talairach coordinates for peak voxel activations after a False Discovery Rate (FDR) correction (*p* < 0.05; minimum size of each cluster was 25 contingent voxels). Indented rows indicate subordinate clusters. Hemispheres: R (Right) or L (Left); Abbreviations: BA, Brodmann Area; G, Gyrus; L, Lobule*.

**Table 4:** **Maxima of activation beginning with the onset of rule cues versus position cues**.

Anatomical area	Cluster size	Hemisphere	*x*	*y*	*z*	*Z* value
**RULE CUE > POS CUE**						
Middle frontal G. (BA 9)	80	L	−35	21	30	4.34
Middle frontal G. (BA 10)		L	−37	37	18	3.49
Precentral G. (BA 9)	28	R	38	23	37	3.49
Middle frontal G. (BA 9)		R	32	33	24	3.40
Inferior frontal G. (BA 44)	270	L	−51	9	15	4.88
Claustrum		L	−29	18	5	4.71
Insula		L	−37	13	2	4.03
Claustrum	93	R	30	18	4	3.97
Putamen		R	21	10	3	3.93
Insula		R	38	18	7	3.90
Pre-SMA (BA 6)	60	L	−10	−2	66	3.71
Superior frontal G. (BA 6)		L	−4	−9	52	3.25
Middle temporal G. (BA 21)	56	L	−51	−41	5	3.56
Middle temporal G. (BA 22)		L	−54	−52	4	3.32
Inferior parietal L. (BA 40)	98	R	37	−54	43	4.74
Inferior parietal L. (BA 40)	475	L	−41	−50	42	5.86
Supramarginal G. (BA 40)		L	−54	−46	26	4.23
Precuneus (BA 7)	41	L	−10	−72	35	3.68
**POS CUE > RULE CUE**						
Anterior cingulate (BA 24)	650	L	−9	33	−1	4.00
Medial frontal G. (BA 9)		R	5	45	14	3.95
Medial frontal G. (BA 9)		L	−12	44	22	3.90
Superior frontal G. (BA 8)	145	R	23	27	51	4.60
Middle frontal G. (BA 8)		R	24	14	41	3.80
Superior frontal G. (BA 6)		R	12	33	51	3.11
Media frontal G. (BA 32)	61	L	−21	12	40	4.60
Posterior cingulate (BA 23)	663	R	7	−57	18	4.76
Precuneus (BA 7)		R	1	−48	43	4.61
Posterior cingulate (BA 30)		L	−15	−53	12	4.54
Middle temporal G. (BA 39)	69	R	48	−66	26	3.91
Middle occipital G. (BA 18)	458	R	26	−84	5	4.52
Cerebellum (declive)		R	27	−63	−9	4.40
Lingual G. (BA 18)		R	29	−74	−8	4.37
Cerebellum (declive)	382	L	−18	−84	−15	4.09
Fusiform G. (BA 19)		L	−29	−74	−11	4.07
Middle Occipital G. (BA 18)		L	−24	−86	1	3.80

*Results are reported in Talairach coordinates for peak voxel activations after a False Discovery Rate (FDR) correction (*p* < 0.05; minimum size of each cluster was 25 contingent voxels). Indented rows indicate subordinate clusters. Hemispheres: R (Right) or L (Left); Abbreviations: BA, Brodmann Area; G, Gyrus; L, Lobule*.

#### RULE cue > NON-cue

**Figure 6:**
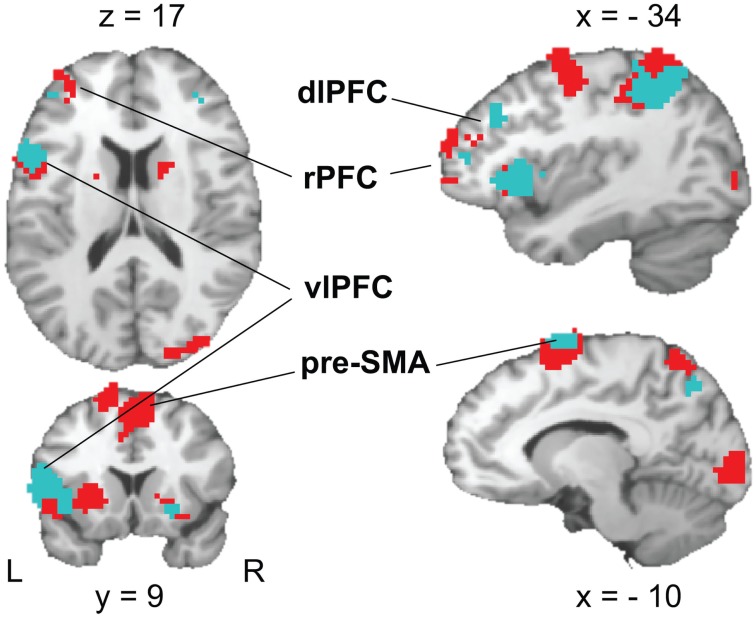
**Activation patterns in brain areas associated with rule cue-induced pre-target processes**. A FDR-corrected (*p* < 0.05, *k* ≥ 25 voxels) T-maps contrasting RULE > NON (red color) and RULE > POS (cyan color, superimposed) are plotted on a single subject Colin brain in MNI space (highlighted areas of interest correspond to Talairach peak voxel coordinates in Tables 2 and 4).

The contrast of *RULE cue* > *NON-cue* highlighted a widespread fronto-posterior network of RULE cue-induced anticipation including frontal areas such as the left lateral rostral prefrontal cortex (rPFC), left posterior vlPFC, left dorsolateral prefrontal cortex (dlPFC), and the pre-SMA (see Figure 6; Table 2 for a complete list that contains also temporal, occipital, basal ganglia, and thalamic activation). The reversed contrast *NON-cue* > *RULE cue* involved the medial rPFC, right dlPFC, and the ACC among other activations (see Table 2).

We tested whether the individual fMRI activation in the frontal areas resulting from the whole brain *RULE cue* > *NON-cue* contrast correlated with the individual RULE cue-induced behavioral benefit (calculated as the *Z*-standardized individual difference between RTs for NON-cues minus RTs for RULE cues). In fact, the success of RULE cue implementation correlated with the cluster activation of the pre-SMA (Talairach coordinates: 7, 5, 53), *r* = 0.38, *p* < 0.05, and the vlPFC (Talairach coordinates: −46, 0, 17), *r* = 0.42, *p* < 0.05, while no such correlations were found for rPFC and dlPFC.

#### POS cue > NON-cue

In contrast to the activation pattern revealed for RULE cue > NON-cue contrast, *POS cues* activated premotor regions and bilateral regions of posterior cortex and occipital cortex. No activated regions were found in the reversed *NON-cue* > *POS cue* contrast (Table 3).

#### RULE cue > POS cue

Like in the RULE > NON-contrast, areas more activated in RULE cues than in POS cues involved the left lateral rPFC, left posterior vlPFC, left dlPFC, and the pre-SMA (Figure 6; Table 4). As can be seen in Table 4, the activated neural network also involves temporal and parietal areas as well as basal ganglia and the insula. The reversed contrast, POS cue > RULE cue revealed anterior and posterior cingular and bilateral dlPFC activation among other premotor, motor, temporal, parietal, occipital, and cerebellar regions.

#### RULE incompatible > RULE compatible

No effect of anticipated compatibility was obtained for this or the reversed contrast.

## General Discussion

The present study aimed at investigating the neural underpinnings of anticipation of action selection within a conflict task. To this end, EEG and fMRI measures were recorded in a Simon task combined with cues that predicted compatibility (rule cues), stimulus location (position cues), or were non-informative. Behavioral results established that rule and position cues were utilized for both compatible and incompatible assignments. Compatible assignments were faster accomplished than incompatible assignments in all cue conditions. Rule cues yielded the strongest benefit for RTs and accuracy. Position cues entailed faster responses than non-informative cues for compatible and incompatible trials as well, although these cue benefits were smaller than those of rule cues. Moreover, accuracy as a function of response time (CAFs) measured response priming by the irrelevant stimulus location (response capture) was strongest after non-informative cues, intermediate after position cues, and weakest after rule cues. Rule cues, therefore, seemed to lead to anticipatory adjustments that prevented response capture. As a measure of anticipatory processes the CNV was largest with rule cues. The post-target N2 indicates online action control (Folstein and van Petten, 2008) that was reduced by rule cues as compared to the other cue conditions. Both rule and position cues canceled out the difference between incompatible and compatible assignments in the conflict-related N2. Non-informative cues were the only condition that generated a compatibility effect in the N2. This N2 result resembles our behavioral accuracy data that also exhibited a compatibility effect solely after non-informative cues and in addition indicated a particularly enhanced response capture in distributional analyses. Taken together, these effects suggest the existence of cognitive conflict after non-informative cues. Viewed in this light, it is questionable whether compatibility effects after rule cues and position cues can be seen as typical Simon or conflict effects. For both informative cues the entire data pattern does not show any conflict-specific pre- or post-target effects besides the behavioral difference in response speed between the compatibility conditions. The overall short RTs and high accuracy (even in the fastest segment of response times) suggest that rule cues considerably simplified the task especially for compatible trials. Rule cues apparently provoked anticipatory adjustments that circumvented conflict in upcoming Simon trials. In our paradigm, rule cues reduced the task set from four to two possible stimulus-response (S-R) assignments. In particular, with rule cues participants knew whether to respond on the same or opposite direction of the stimulus location. Participants might thus have translated the instruction into more effective condition-action rules by first excluding NoGo trials and then responding according to the target position. Wühr and Kunde (2008) have previously shown that participants use such re-coding to circumvent conflict in two-choice Simon task. In fact, we introduced NoGos to get a three-choice task in order to avoid such a task reconfiguration. However, this manipulation was most probably not effective. This reasonable suspicion was further supported because false alarms occurred more often for NoGos after rule cues as compared to the other cue conditions indicating a higher readiness to respond after rule cues. The RT difference between compatible and incompatible Simon trials after rule cues may as such not be due to Simon conflict but might simply come about by differences in response selection complexity. In incompatible trials after rule cues one has to respond opposite to stimulus location which is a more complex response selection rule than selecting the response according to stimulus position in compatible trials. In a similar vein, position cues minimized the uncertainty about the stimulus position and allowed to anticipate the upcoming target location. Anticipatory attentional allocation to the target position might have reduced the cause for the Simon conflict, namely spatial uncertainty. However, position cues did not allow for a condition-action rule remapping like rule cues, because choice responses were still due to stimulus figure which was not known in advance. The latter fact might explain why the numerical reduction of the N2 induced by position cues against non-informative cues failed significance.

In contrast to present findings rule cues accelerated only compatible not incompatible assignments in our former study (Alpay et al., 2009). Two possible reasons can account for this difference: first, the 1.5-s cue-target interval in the former study (as compared to 6 s in the present study) may have been insufficient for preemptive anticipatory regulation in incompatible Simon trials, which need a translation into a more complex response selection rules as compared to compatible trials. Second, the complexity of the translation may have been additionally aggravated by the vertical design in the former study as compared to the more natural horizontal design that relates to bilateral body and brain symmetries in the present study (Vallesi et al., 2005). Most probably for the same reasons, our former study could also not reveal any rule cue-specific CNV modulation (Alpay et al., 2009) which we clearly observed in the present study.

The fMRI results indicated differences between anticipatory processes triggered by rule and position cues. A key finding was that rule cues induced more activation of the left lateral rPFC, left posterior vlPFC, left dlPFC, and the pre-SMA as compared to both position and non-informative cues. The rPFC has been proposed to enable other prefrontal regions to assist whenever rules have to be elaborated on a higher order level or when task management is in demand (Koechlin et al., 1999; Christoff and Gabrieli, 2000; Sakai and Passingham, 2003, 2006; Ramnani and Owen, 2004; Badre and D’Esposito, 2007; Wolfensteller and von Cramon, 2010, 2011). In the present study, rule cues were associated with more lateral rPFC activation than non-informative cues while the reversed contrast involved medial rPFC activation. Lateral activations of the rPFC are mostly associated with the maintenance and/or retrieval of task-relevant information while medial activation are present in studies investigating internal attending (Gilbert et al., 2006, for a meta-analysis). Rule-based response selection, especially when the task involves inhibitory or complex rules, has been consistently related to dlPFC activation (Sakai and Passingham, 2003; Bunge and Souza, 2008). Some studies also report anticipatory dlPFC activation (Fassbender et al., 2006; Luks et al., 2007) although the conflict monitoring theory posits that it is the resolution of cognitive conflict that takes place in the dlPFC (Botvinick et al., 2001).

Interestingly, the pre-SMA and vlPFC activation correlated with the behavioral benefit participants derived from rule cues. This finding points to the importance of these areas for the effective preparation of specific response contingencies. The left posterior vlPFC and pre-SMA have been previously associated with the maintenance of task-relevant knowledge that is used to guide subsequent behavior (for a review, see Bunge, 2004). Participants can mentally rehearse response contingencies using phonological codes while they can also prepare to respond with one or more effectors, by maintaining relevant high-level (i.e., relatively abstract) action representations (Bunge, 2004). Phonological rehearsal during task preparation has been claimed to be associated with activation of the left posterior vlPFC (Smith and Jonides, 1999; Bunge et al., 2003; Wagner et al., 2004) while abstract action representation rather involves the pre-SMA (Hazeltine et al., 2000). It is plausible that participants internally rehearsed their re-coded rules and at the same time maintained associated action codes in the present study. According to previous studies, pre-SMA activation seems to be involved in voluntary prospective action control (Sumner et al., 2007) representing action intentions (Lau et al., 2004) and initiating action sequences (Kennerley et al., 2004) rather than actual movements. As well, the pre-SMA is involved when S-R associations have to be re-learned or reversed (Nakamura et al., 1998), and when response competition is present (Milham et al., 2001; Derrfuss et al., 2004; Kennerley et al., 2004; Nachev et al., 2007; Taylor et al., 2007). Recently, it has been discussed whether the pre-SMA might be associated with prospective anticipatory regulation by selectively preparing the appropriate task set and triggering adaptation to conflict (Hikosaka and Isoda, 2010; King et al., 2010; Ullsperger and King, 2010). Importantly, the present data do not necessarily suggest a specific role of the pre-SMA and vlPFC in conflict control but rather indicate that these areas might be activated whenever prospective task reconfigurations are applied in order to reduce complexity or computational load during task implementation.

Furthermore, no indications of compatibility-specific processes within rule cues were found in the present study. The anticipation of an incompatible trial did not differ from the anticipation of a compatible trial (see also Forstmann et al., 2008b, for similar results in a Simon task fMRI study). This holds not only true for CNV and fMRI measures in the present study but also for the CNV results in our former study (Alpay et al., 2009). As mentioned before, participants re-coded the task by shifting their attentional focus on target position instead of target figure; hence the absence of anticipatory conflict-specific processes may not be surprising. It can rather be seen as an additional evidence for how effectively such preemptive mechanisms can prevent potentially effortful situations. The few studies that previously investigated anticipatory high-conflict versus low conflict effects mostly focused on predictions based on the conflict monitoring account. Some found anticipatory high versus low conflict ACC activation while others did not (Fassbender et al., 2006; Luks et al., 2007; Sohn et al., 2007; Aarts et al., 2008; Donohue et al., 2008). A reasonable explanation for these disparate findings is that anticipatory action regulation can be accomplished through different types of anticipatory adjustments. In particular, high versus low conflict conditions may trigger different usage of proactive or preemptive adjustments depending on the costs and benefits in terms of cognitive effort. In a similar vein prospective anticipatory adjustments are investigated in the field of task switching. A recent review of brain networks accomplishing task preparation showed a heterogeneous pattern of brain areas related to anticipatory regulation across studies (Ruge et al., 2013). The authors argue for different preparatory regulation modes that, e.g., focus on action-related or attention-related sets. The key for a better understanding of anticipatory control may lie in a careful separation of the actual underlying regulatory processes such as preemptive and proactive adjustments.

## Conflict of Interest Statement

The authors declare that the research was conducted in the absence of any commercial or financial relationships that could be construed as a potential conflict of interest.

## Footnotes

^1^Note that the term “proactive” has a different connotation in the dual routes framework of Braver et al. (2007).

^2^Our previous study showed that NoGo-related processes did not modulate behavioral and electrophysiological cueing and Simon effects Alpay et al. (2009).

^3^www.neurobs.com

^4^www.fil.ion.ucl.ac.uk

^5^http://imaging.mrc-cbu.cam.ac.uk/imaging/MniTalairach
